# Neutralisation of uPA with a Monoclonal Antibody Reduces Plasmin Formation and Delays Skin Wound Healing in tPA-Deficient Mice

**DOI:** 10.1371/journal.pone.0012746

**Published:** 2010-09-15

**Authors:** Annika Jögi, Birgitte Rønø, Ida K. Lund, Boye S. Nielsen, Michael Ploug, Gunilla Høyer-Hansen, John Rømer, Leif R. Lund

**Affiliations:** Finsen Laboratory, Copenhagen University Hospital, Copenhagen Biocenter, Copenhagen, Denmark; Brunel University, United Kingdom

## Abstract

**Background:**

Proteolytic degradation by plasmin and metalloproteinases is essential for epidermal regeneration in skin wound healing. Plasminogen deficient mice have severely delayed wound closure as have mice simultaneously lacking the two plasminogen activators, urokinase-type plasminogen activator (uPA) and tissue-type plasminogen activator (tPA). In contrast, individual genetic deficiencies in either uPA or tPA lead to wound healing kinetics with no or only slightly delayed closure of skin wounds.

**Methodology/Principal Findings:**

To evaluate the therapeutic potential in vivo of a murine neutralizing antibody directed against mouse uPA we investigated the efficacy in skin wound healing of tPA-deficient mice. Systemic administration of the anti-mouse uPA monoclonal antibody, mU1, to tPA-deficient mice caused a dose-dependent delay of skin wound closure almost similar to the delayed kinetics observed in uPA;tPA double-deficient mice. Analysis of wound extracts showed diminished levels of plasmin in the mU1-treated tPA-deficent mice. Immunohistochemistry revealed that fibrin accumulated in the wounds of such mU1-treated tPA-deficent mice and that keratinocyte tongues were aberrant. Together these abnormalities lead to compromised epidermal closure.

**Conclusions/Significance:**

Our findings demonstrate that inhibition of uPA activity with a monoclonal antibody in adult tPA-deficient mice mimics the effect of simultaneous genetic ablation of uPA and tPA. Thus, application of the murine inhibitory mU1 antibody provides a new and highly versatile tool to interfere with uPA-activity in vivo in mouse models of disease.

## Introduction

Tissue remodeling and confined degradation of the extracellular matrix (ECM) is pivotal in several physiological and pathological processes involving cell migration [1]–[5]. This tightly controlled proteolytic degradation of the ECM is mainly performed by the serine protease plasmin and members of the matrix metalloproteinase (MMP) family [3], [6]. Plasmin is synthesized as a precursor, plasminogen (Plg), in the liver, and is present throughout the body in micromolar concentrations. Plg is activated at its site of action by proteolytical cleavage by one of three proteases, urokinase-type plasminogen activator (uPA), tissue-type plasminogen activator (tPA) [7], [8,] or the newly identified Plg activator, plasma kallikrein [9]. Plg deficiency has severe physiological consequences, primarily due to diminished fibrinolysis, in both humans and mice [10]–[12]. Furthermore, gene disruption studies in mice have proven plasmin(ogen) to be required for the proper execution of processes involving ECM remodeling, such as cancer metastasis [13], neointima formation after vascular injury [14], placental development [15], post-lactational mammary gland involution [16], and skin wound healing [17]. In Plg-deficient mice there is a marked delay in healing of incisional skin wounds, presumably due to a diminished ability of the leading-edge keratinocytes at the wound edges to proteolytically dissect their way through the fibrin-rich wound matrix, as fibrin is accumulating around these keratinocytes [17]. The previous finding that lack of fibrin(ogen) in the wound field rescues the requirement for Plg to achieve timely healing [18] further corroborates that the primary role for Plg in wound healing is fibrinolysis. In addition, we have recently demonstrated that Plg activation in wounds is actually dependent on the presence of this fibrin-rich provisional matrix [19].

During the invasive phase of wound healing, the migrating leading-edge keratinocytes express uPA and its cell surface receptor uPAR [20], [21], whereas tPA has been detected only in a few keratinocytes late in the re-epithelialization of human wounds [20]–[22]. In addition to the expression of components of the Plg activation system, several members of the MMP family, including MMP-3, MMP-9 and MMP-13, are expressed in the leading-edge keratinocytes in mice [23], [24]. The physiological process, whereby keratinocytes detach from the epithelium and invade into the wound matrix during the healing process, has been described as epithelial to mesenchymal transition with many similarities to the pathological process of tumor invasion and metastasis (for overview see [25]). This suggests that wound healing can be used as a model system for studies of cancer cell invasion (for reviews see [5], [26]).

Recently, it was demonstrated that systemic administration of an anti-catalytic monoclonal antibody (mAb) against uPA (termed mU1) rescues mice treated with an otherwise lethal dose of a uPA activity-dependent bacterial pro-toxin and that it successfully impairs uPA-mediated fibrinolysis in tPA-deficient mice [27]. Targeting a protease with an inhibitory antibody provides an opportunity to study tissue remodeling processes in adult mice in a well-defined time period as opposed to gene targeting approaches.

We have previously demonstrated that mice double-deficient for both uPA and tPA have a prolonged mean healing time in a full-thickness incisional skin wound model compared to wild type mice [28], [29]. In the present study, we provide evidence that systemic treatment with the neutralizing mAb mU1 [27] delays wound healing in tPA-deficient mice in a dose-dependent manner.

## Materials and Methods

### Animals and animal treatment

All breeding and experimental procedures took place at the Department of Experimental Medicine, Copenhagen University, Denmark and were performed according to institutional and national guidelines and approved by the Danish Animal Experiments Inspectorate (2005/561-1014). tPA-deficient mice [30] were backcrossed to C57Bl/6J mice for 22 generations, and used for breeding of heterozygous parents that yielded gene-deficient and wild type littermates. uPA;tPA double-deficient mice were obtained by intercrosses of double heterozygous uPA^−/+^;tPA^−/+^ mice as described previously [29]. All mice in this study were 6–8 weeks old at the start of experiments.

**Figure 1:**
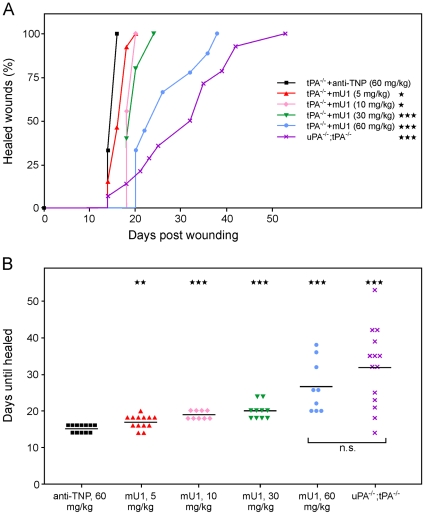
Delayed wound healing in tPA-deficient mice after systemic treatment with a monoclonal anti-uPA antibody, mU1. A, The percentage of mU1-treated and anti-TNP-treated tPA-deficient (tPA^−/−^) mice as well as uPA;tPA double-deficient (uPA^−/−^;tPA^−/−^) mice with wounds that have healed completely are depicted in a Kaplan-Meier plot, n = 9–14. B, The mean healing time with increasing doses of mU1 administration to tPA-deficient mice compared to control mAb-treated (anti-TNP) as well as to uPA;tPA double-deficient (uPA^−/−^;tPA^−/−^) mice. Mann-Whitney unpaired t-test was applied to statistically compare healing of mU1-treated and control mAb (anti-TNP)-treated mice. *** indicates p-values, i.e. * p<0.05, ** p<0.005, and ***p<0.0001.

Full-thickness incisional skin wounds were made, measured over time, and collected for histological analysis as described previously [24]. Briefly, mice were anaesthetized with a mixture of Fentanyl, Dormicum, and Midazolam, shaved on the back, and a 20 mm full-thickness skin incision was made exactly along the back midline. The length and width of the wounds were measured every second day until two successive measurements were considered fully healed, i.e. loss of the wound scab and complete re-epithelialization. Previous studies revealed that this is an easy, robust, and reproducible method for analysis of overall healing of large insicional wound [29]. Statistical analysis of wound length and healing time were performed using GraphPad Prism 3.0 and Kaplan-Meier analysis (Figure 1A) Mann-Whitney test (Figure 1B). The null hypothesis was rejected when the P-value was less than 0.05. Wound tissue from the mice given the highest antibody dose were collected for histology on day 7, day 10 and day 21 after wounding (3 mice per time-point). Mice were anaesthetized as described above and perfused by intracardial injection of 10 mL cold phosphate buffered saline and 10 mL 4% paraformaldehyde in phosphate buffered saline. The wound area and adjacent tissue was dissected and fixed over night in 4% paraformaldehyde, dehydrated and subsequently embedded in paraffin.

The mouse mAb against mouse uPA, termed mU1 [27], or anti-trinitrophenyl (anti-TNP) [31], which was used as an IgG1 subtype-matched control antibody, were injected intraperitoneally into tPA-deficient mice and wild type siblings. Doses were 5 (13 mice), 10 (9 mice), 30 (10 mice), or 60 mg/kg (9 mice, plus 9 mice for histology) of mU1 and 60 mg/kg (12 mice, plus 9 mice for histology) of anti-TNP per mU1 half-life (3.2 days) [27]. The injections were given once (5 mg/kg and 10 mg/kg) or twice (due to larger injection volumes, 30 and 60 mg/kg) weekly until the wounds were fully re-epithelialized and the scab came off. To reach steady-state levels of the antibody in question an initial dose of twice the maintaining dose was given 4 to 24 hours before wounding. For comparision 14 tPA;uPA double deficient mice were included, these were not given any antibody. Injections, measurement of wound length, tissue preparation, and analyses were performed by persons unaware of the treatment and the genotype of the mice.

### Production and purification of monoclonal antibodies

The mAb against mouse uPA, mU1, was generated by immunization of uPA-deficient mice with a recombinant pro-form of mouse uPA [27]. Large-scale production and purification of mAb mU1 and anti-TNP has previously been described [32]. The mAbs were routinely analyzed for endotoxin levels using the Limulus Amebocyte QCL-1000 lysate method (Bio Whittaker, MD, USA), and always found to be below 0.4 EU/g mouse per injection.

### Histological analysis and immunofluorescence examination

Paraffin-embedded wounds obtained from untreated tPA-deficient and uPA;tPA double- deficient mice as well as from tPA deficient mice, which had been treated with either 60 mg/kg mU1 or control mAb twice a week for 7 or 21 days post wounding, were sectioned and stained according to standard procedures. Immunofluorescence stainings of fibrin by a rabbit anti-mouse fibrin(ogen) antibody [10] (1∶2000) and of cytokeratin by a polyclonal goat antibody (1∶500, ab 8572; Abcam, Cambridge, USA), were performed essentially as previously described [29], [32]. Acquisition was performed with a Leica DM4000B microscope using a Leica 10× magnification lens.

### Wound extracts and immunoblot analysis

In a separate experiment tPA-deficient mice were given either mU1 (n = 9) or anti-TNP (n = 8) (60 mg/kg) and inflicted with a skin wound along the back mid-line, as described above. Wound tissues were harvested from 4 mice from each group 7 days after wounding, and from 5 mU1-treated and 3 control mAb-treated 21 days after wounding (one mouse in the control group was prematurely euthanatized, due to injection failure). Wound extracts from mice treated with either 60 mg/kg mU1 or control mAb were analyzed by immunoblotting, performed as described previously [29]. Briefly, frozen tissue powder of the wound rim and granulation tissue was resuspended in 5 µL of 0.1 M Tris/HCl, pH 7.4, 10 µg/mL aprotinin per mg wet weight of tissue, and subjected to ultrasound for two times 8 minutes. After centrifugation at 12000 rcf for 30 minutes the supernatants were collected. Samples were separated electrophoretically under reduced condition and electroblotted onto PVDF membranes. Plasminogen/plasmin was detected using polyclonal rabbit anti-human plasminogen antibody (DAKO A0081, diluted to 0.5 µg/mL) and HRP-linked swine anti-rabbit antibody (DAKO, P0217) as previously described [29].

## Results

### Dose-dependent delay in wound healing after systemic treatment with mU1

Cohorts of tPA-deficient mice received mU1 intraperitoneally at the doses 5 (13 mice), 10 (9 mice), 30 (10 mice), or 60 mg/kg (9 mice) per mU1 half-life, or an IgG subtype control mAb of irrelevant specificity (anti-TNP) 60 mg/kg (12 mice). The half-life of mU1 in vivo was previously determined to be approximately 3 days [27]. For comparison uPA;tPA double-deficient mice were included (14 mice). Administration of mU1 to the tPA-deficient mice caused a dose-dependent increase in the healing time of the incisional skin wounds, measured as the time to complete re-epithelialization (Figure 1A and B). Since the healing times for mU1-treated mice at the highest dose (i.e. 60 mg/kg) approached that of uPA;tPA double-deficient mice (Figure 1A), and only mU1-treated animals displayed a significant increase in the mean healing times compared to control mAb-treated tPA-deficient mice, the delay in healing was caused by in vivo inhibition of uPA activity by mU1 and not an effect of IgG1 administration. There was no statistically significant difference in mean healing time between tPA-deficient mice given the highest dose of mU1 and the mock-treated uPA;tPA double-deficient mice (p = 0.27 Mann-Whitney unpaired t-test; Figure 1B). Importantly, mU1 administration to wild type mice did not result in any significant delay in healing time (data not shown), which is in accordance with the healing kinetics observed in uPA-deficient mice [29].

### Plasminogen activation is decreased in wound extracts from mU1-treated tPA-deficient mice

**Figure 2:**
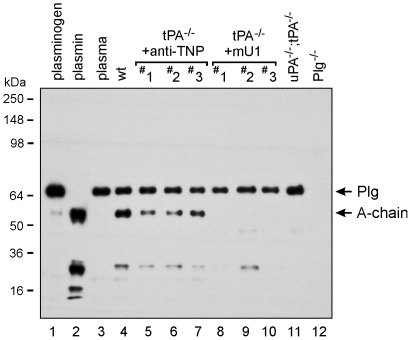
Immunoblot analysis for plg/plasmin in wound extracts. Immunoblot assay with plasminogen (lane 1), plasmin (lane 2), murine plasma (lane 3), and wound extracts 7 days post wounding obtained from wild type mice (wt, lane 4), control mAb-treated (i.e. anti-TNP) tPA-deficient mice (lanes 5–7), mU1-treated tPA-deficient mice (lanes 8–10), uPA;tPA double-deficient mice (lane 11), and Plg-deficient mice (lane 12). Plasminogen and plasmin were detected using a polyclonal rabbit anti-human plasminogen antibody. #1, #2, #3 denote wound extracts from three different mice.

We next addressed whether systemic treatment with mU1 affected plasminogen activation in skin wounds by analyzing the proteins in skin wound lysates by immunoblotting using a polyclonal antibody that recognizes both plasminogen and plasmin [29]. We found reduced levels of plasmin in extracts prepared from 7 days old wounds in mU1-treated mice, compared to time-matched wound extracts from control mAb-treated tPA-deficient mice (Figure 2, lanes 8–10 versus lanes 5–7). The plasmin levels in the mU1-treated tPA-deficient mice was almost undetectable, hence resembling the levels in uPA;tPA double-deficient mice (Figure 2, lanes 8–10 versus lane 11), in which plasmin is known only to be detectable in wound extracts after a purification step [29]. This result clearly demonstrates that systemic administration of the inhibitory mU1 mAb reduces plasminogen activation in vivo.

### Excessive fibrin accumulation in skin wounds from mU1-treated tPA-deficient mice

**Figure 3:**
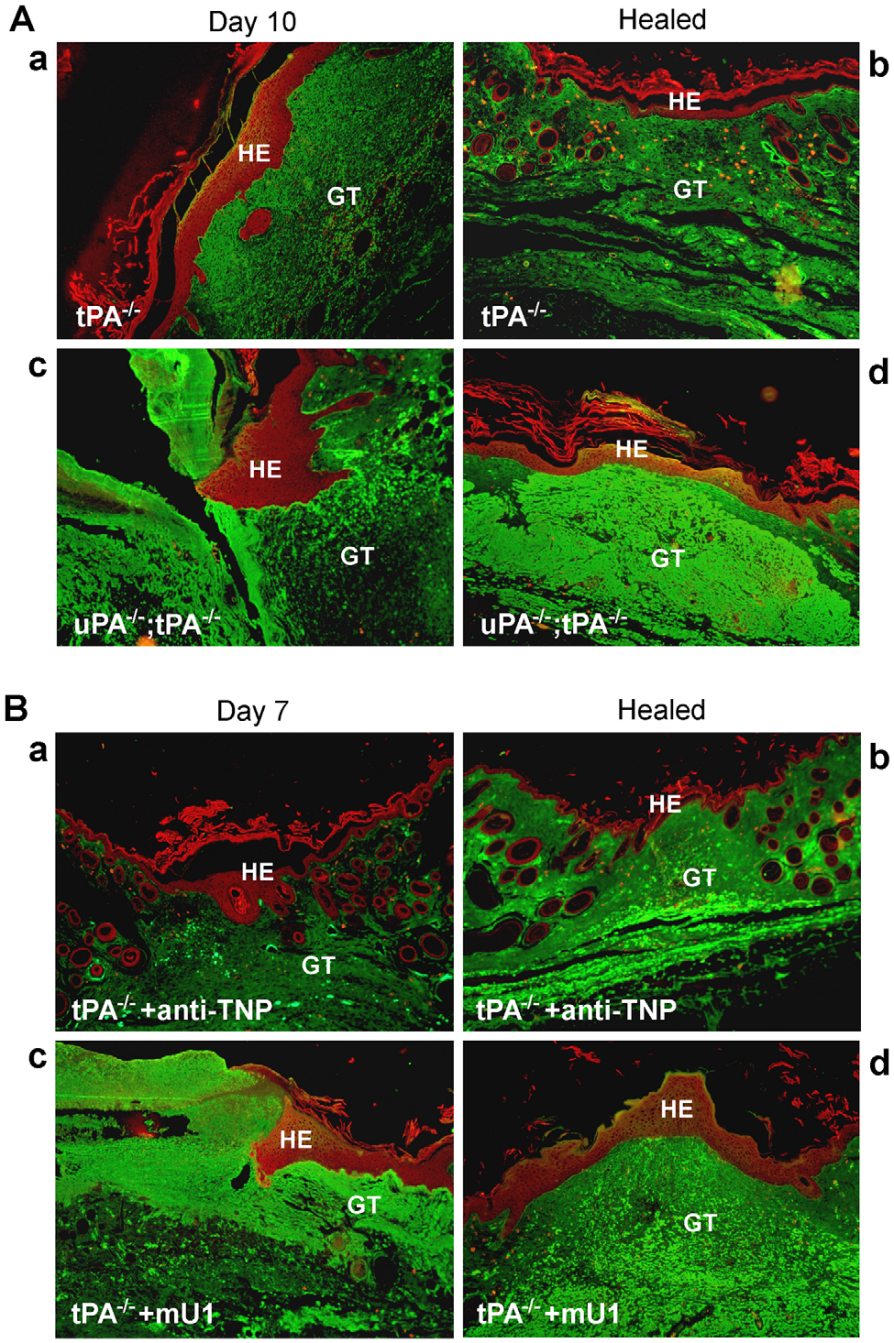
Cytokeratin and fibrin immunofluorescence stainings of wound areas from mU1- or control mAb (i.e. anti-TNP)-treated mice. Double immunofluorescence staining of cytokeratin (546 nm, red) and fibrin/fibrinogen (488 nm, green) in wound sections. A, Wounds from tPA-deficient and uPA;tPA double-deficient mice during healing (left panel, 10 days post wounding) and after re-epithelialization (right panel). B, Micrographs of wounds isolated from control mAb-treated (upper panels) and mU1-treated (lower panels) mice during healing (left panel, 7 days post wounding) and upon healing (right panel, 21 days post wounding).HE; hyperproliferative epidermis, GT; granulation tissue.

uPA;tPA double-deficient mice displayed excess fibrin deposition in the wound area, both during healing and upon complete re-epithelialization, as demonstrated by fibrin and pankeratin double immunofluorescence staining (Figure 3A, panel c and d). Only a moderate amount of fibrin was present in the wound area in tPA-deficient mice, both 10 days post wounding and after healing (Figure 3A, panel c and d).

In a separate experiment tPA-deficient mice were treated with either mU1 (n = 9) or anti-TNP (n = 8) (60 mg/kg) and inflicted with a skin wound along the back mid-line, as described above. Wounds were harvested from 4 mice from each group 7 days post wounding, and from 5 mU1-treated and 3 control mAb-treated 21 days post wounding (one mouse in the control group was prematurely euthanatized, due to injection failure). Treatment of tPA-deficient mice with mU1 resulted in accumulation of excess fibrin in the wound area as compared to control mAb-treated tPA-deficient mice at day 7 post wounding (Figure 3B, panel a versus c). Twenty-one days post wounding, the wounds of all control mAb-treated and one out of 4 of the mU1-treated mice were completely re-epithelialized. The remaining 4 mU1-treated mice had in mean 6 mm remaining of the originally 20 mm wound (4.5–7 mm). In the mU1-treated animals more fibrin immunostained material was detected throughout the dermal tissue in the wound area compared to control mAb-treated (Figure 3B, panel b versus d). This may in part reflect that the wounds in mU1-treated mice, at this time point 21 days after wounding, were fully closed. However, it also demonstrates that in vivo neutralization of uPA activity by mU1 leads to impaired fibrin degradation.

## Discussion

Delayed healing of skin wounds is a well-characterized phenotype of uPA;tPA double-deficient mice [28], [29]. In order to obtain acute disruption of uPA activity, we used a neutralizing monoclonal antibody directed against murine uPA, mU1, in a thoroughly characterized wound-healing model [19], [29], where a 20 mm full thickness incisional skin wound is inflicted along the back midline of an anaesthetized mouse. Endpoints were wound lengths measured every second day and time to complete wound closure defined as seamless epidermal regeneration and loss of the wound scab. We have previously shown that mU1 is capable of inhibiting uPA-mediated plasminogen activation without interfering with uPA receptor binding in vitro as well as in vivo. Furthermore, we have demonstrated that mU1-treatment of tPA-deficient mice causes hepatic fibrin accumulation, recapitulating the observation in the gene-targeted uPA;tPA double-deficient mice [27]. These effects of the anti-uPA mAb treatment were significant as compared to the control treatment, while at the same time being statistically indistinguishable from, though not completely identical to the phenotype found in the uPA;tPA double-deficient mice. One explanation for this could be that in the gene-targeted mouse the protein is permanently absent, while in the mU1-treated mouse it is present and only the catalytic activity is abrogated by mU1. It has additionally been reported that uPA can stimulate a chemotactic response, which is independent on the catalytic activity and only relies on the presence of the amino-terminal part of uPA binding to uPAR [33]. If both the catalytic activity and the chemotactic response are required for wound healing and hepatic fibrin deposition to occur, neutralization of just the catalytic activity will not fully mimic the effect of gene-targeting. The less than complete effect of mU1 may be explained by fluctuating plasma levels of the mAb, as proposed by others taking this approach for in vivo targeting of specific proteins [34]. Since we have previously demonstrated that treatment with a mAb directed against murine uPAR resulted in a phenotype fully mimicking the effect observed in the gene-targeted mouse with respect to hepatic fibrin accumulation [32], specificity and affinity of the antibody itself may also influence the outcome.

We conclude that systemic inhibition of uPA enzymatic activity by a monoclonal antibody in adult mice yields a phenotype that resembles that of the gene-targeted mice. The observed delay in wound healing of these tPA-deficient mice can thus be attributed to the plasminogen activating effect of uPA and not to the effects elicited by the uPA-uPAR association per se, as this complex is not disturbed by the mU1 antibody [27]. The specific sites and situations of fibrin accumulation in uPA/uPAR targeted and uPA;tPA double-deficient mice point at the redundancy of fibrin clearance in the physiological situation in most organs, whereas in specific processes, i.e. wound healing, and organs, in particular the liver, the balance between fibrin deposition and clearance is more delicate and the effect of diminished plasminogen activation is revealed. The deposition of excess fibrin in the healing wounds in mU1-treated tPA-deficient mice is in agreement with our previous finding that mU1-treated tPA-deficient mice are not able to clear fibrin from the hepatic sinusoids [27]. Accumulation of fibrin in the liver also occurs in tPA-deficient mice treated with a monoclonal antibody against uPAR, and in these mice the hepatic fibrin plaques were shown to hold uPAR-expressing macrophages, indicating a role for hepatic macrophages in fibrin-clearance [32].

Prognostic and predictive studies have demonstrated the importance of uPA in cancer [35] and uPA is expressed at the invasive front by stromal cells in human breast and colon cancer [36], [37]. Several poly- and monoclonal antibodies as well as peptide antagonists have been generated and tested in different xenografted tumor models. However, they all target the proteins of the human plasminogen activation cascade, making them insufficient as tools in preclinical mouse models, where the roles of host-derived proteases are crucial [6]. The migration and proliferation of keratinocytes during wound closure have similarities to cancer invasion and is thus employed to model this event. Conclusively, systemic treatment with anti-uPA monoclonal antibody, mU1, was effective in delaying wound healing and may thus have a therapeutic potential in mouse cancer models.
